# External Use of Digitalis in Suppression of Urine

**Published:** 1879-08

**Authors:** 


EXTERNAL USE OF DIGITALIS IN SUPPRESSION OF URINE.

Dr. C. P. Russell, in British *Medical Journal.*—A married woman, aged 35, was attacked by acute albuminuria. The disease resisted the usual remedies. She became extremely œdematous, with congestion or œdema of both lungs. Respiration rapid and pulse weak and rapid. She became semi-comatose and there was suppression of urine for 36 hours.

The case appeared hopeless, but having read in the *Journal* of a case in which the external use of digitalis was effectual in restoring the secretion of urine, I determined to try it. I ordered a half ounce of the tincture on a large linseed-meal poultice, to be applied to the abdomen. Next day I was agreeably surprised to find her vastly improved, quite conscious and cheerful. The oedema was very much diminished, respiration was easy and the pulse nearly natural. I was informed, that in one hour after the application, a copious flow of urine commenced and continued all night—and, what was very remarkable, the urine which the day before contained a large quantity of albumen, was now quite free from it. Convalescence was rapid, and she is now quite well.

